# Molecular basis for substrate recognition and septum cleavage by AtlA, the major *N*-acetylglucosaminidase of *Enterococcus faecalis*

**DOI:** 10.1016/j.jbc.2022.101915

**Published:** 2022-04-07

**Authors:** Véronique Roig-Zamboni, Sarah Barelier, Robert Dixon, Nicola F. Galley, Amani Ghanem, Quoc Phong Nguyen, Héloize Cahuzac, Bartłomiej Salamaga, Peter J. Davis, Yves Bourne, Stéphane Mesnage, Florence Vincent

**Affiliations:** 1 CNRS, Aix Marseille University, AFMB, Marseille, France; 2 School of Biosciences, University of Sheffield, Sheffield, United Kingdom

**Keywords:** glycoside hydrolase, structure–function, crystal structure, *Enterococcus*, cell wall, peptidoglycan, AtlAc, catalytic domain of AtlA, BHI, brain heart infusion, GH, glycoside hydrolase, PG, peptidoglycan, Wat12, water molecule 12

## Abstract

The cleavage of septal peptidoglycan at the end of cell division facilitates the separation of the two daughter cells. The hydrolases involved in this process (called autolysins) are potentially lethal enzymes that can cause cell death; their activity, therefore, must be tightly controlled during cell growth. In *Enterococcus faecalis*, the *N*-acetylglucosaminidase AtlA plays a predominant role in cell separation. *atlA* mutants form long cell chains and are significantly less virulent in the zebrafish model of infection. The attenuated virulence of *atlA* mutants is underpinned by a limited dissemination of bacterial chains in the host organism and a more efficient uptake by phagocytes that clear the infection. AtlA has structural homologs in other important pathogens, such as *Listeria monocytogenes* and *Salmonella typhimurium*, and therefore represents an attractive model to design new inhibitors of bacterial pathogenesis. Here, we provide a 1.45 Å crystal structure of the *E. faecalis* AtlA catalytic domain that reveals a closed conformation of a conserved β-hairpin and a complex network of hydrogen bonds that bring two catalytic residues to the ideal distance for an inverting mechanism. Based on the model of the AtlA–substrate complex, we identify key residues critical for substrate recognition and septum cleavage during bacterial growth. We propose that this work will provide useful information for the rational design of specific inhibitors targeting this enterococcal virulence factor and its orthologs in other pathogens.

*Enterococcus faecalis* is a nosocomial pathogen causing a wide range of infections, some of which can be life threatening (1). The virulence of this organism relies on a combination of several factors acting synergistically to outcompete other organisms and cause the disease. These factors include the capacity to survive abiotic stresses (such as pH changes or exposure to bile salts) and antibiotic treatments (*e.g*., cephalosporins) (1, 2). During pathogenesis, *E. faecalis* has evolved several mechanisms that enable this organism to evade the host innate immune system. These include the production of two distinct cell surface polymers (a capsule and a rhamnopolysaccharide) that play an antiphagocytic role (for review, see Ref. (3)). Another mechanism relies on the control of cell chain length. *E. faecalis* has a distinctive morphology and forms diplococci and short cell chains made of four to eight cells. The disruption of cell separation at the end of the division, leading to the formation of cell chains, has a dramatic impact on virulence. It prevents the dissemination of bacteria from causing a systemic infection and leads to the uptake of cell chains by phagocytes (4).

One peptidoglycan (PG) hydrolase, AtlA, has a predominant role in *E. faecalis* septum cleavage. *atlA* mutants are viable but form extremely long chains and are greatly attenuated in the zebrafish virulence model of infection (4). It has also been shown that AtlA activity contributes to the bactericidal activity of beta-lactam antibiotics (5, 6). Based on the contribution of AtlA to pathogenesis and antibiotics-mediated killing, this PG hydrolase represents an attractive target to prevent enterococcal infections whilst limiting microbiota changes.

AtlA is a multimodular *N*-acetylglucosaminidase belonging to the glycoside hydrolase GH73 family in the CAZy (Carbohydrate-Active enZYmes Database) (7). To avoid AtlA-mediated autolysis, the activity of AtlA is tightly controlled, both spatially and temporally (4). The enzyme undergoes post-translational modifications (glycosylation and proteolytic cleavage) and preferentially binds to denuded glycan chains (preferentially found at the midcell) *via* the C-terminal LysM domain (8).

In this study, we report the first structure that captures a closed conformation of the active site indicating a single-displacement (inverting) mechanism. We further provide a structure–function analysis of AtlA catalytic domain. We demonstrate an endoglucosaminidase activity and identify the critical residues for catalysis both *in vitro* and in the context of the live bacteria. Based on these data, we propose a model for the interaction of AtlA with PG, a first step toward the rational design of specific inhibitors targeting this enterococcal virulence factor.

## Results

### Overall structure of the catalytic domain of AtlA

**Figure 1:**
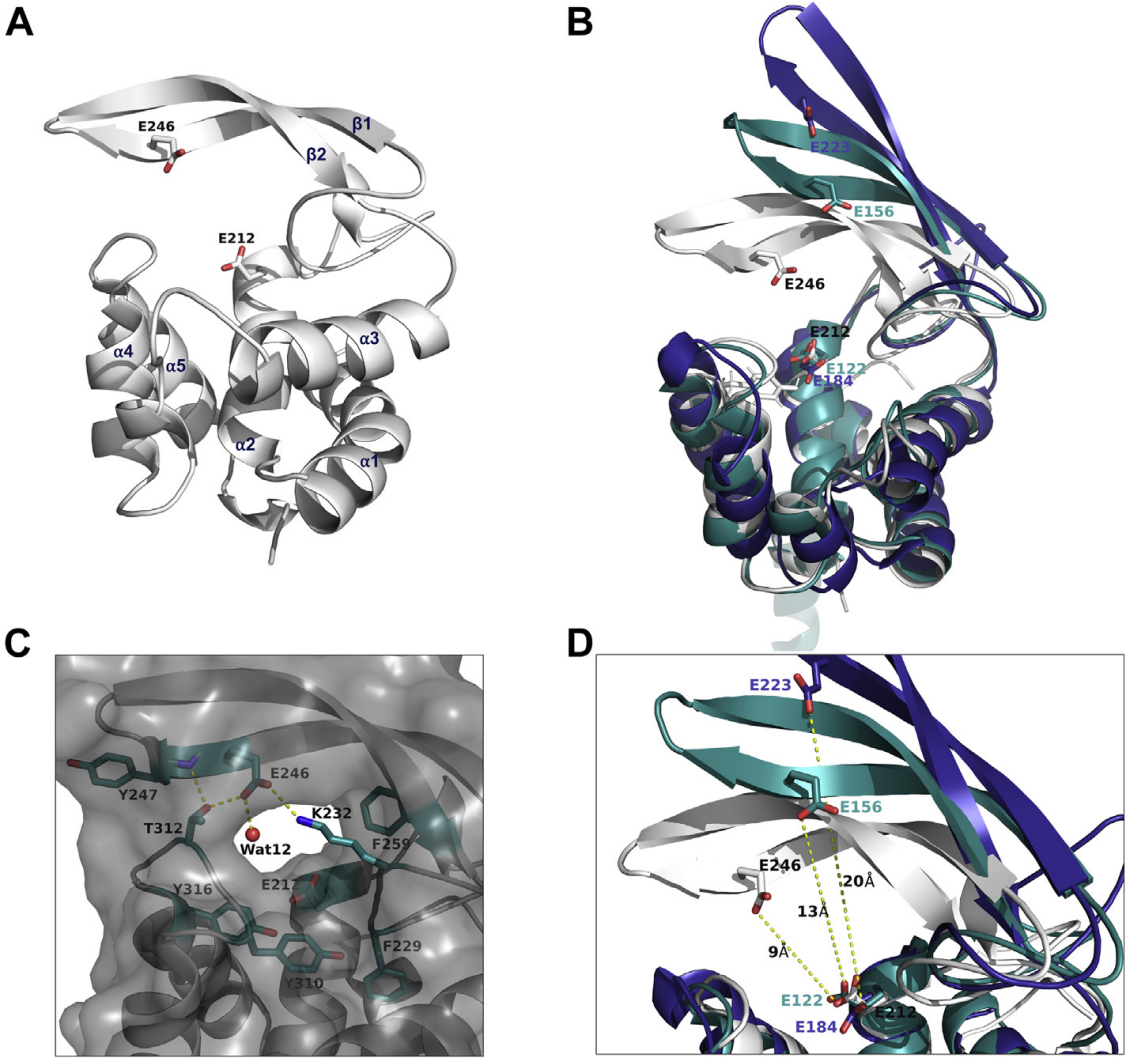
**AtlAc structure and overlay of AtlAc, Auto and FlgJ**_**St**_**.***A*, cartoon representation of the structure of AtlAc. The secondary elements are numbered, and the catalytic E246 and E212 are shown in *stick*. *B*, overlay of AtlAc in *white* (Protein Data Bank [PDB] code: 7QFU), Auto from *Listeria monocytogene* (*blue*, PDB code: 3FI7), and FlgJ from *Salmonella thyphimurium* (FlgJ_St_, *green*, PDB code: 5DN5). The conserved putative catalytic Glu is shown in *stick and colored labels*. *C*, close-up view of the active site; conserved residues are shown in *blue sticks*, and hydrogen bonds are illustrated with *yellow dashed lines*. *D*, comparison of the β-lobe opening in AtlAc (*white*) and Auto (*blue*), the distance between their respective two putative catalytic Glu is shown with *yellow dotted lines*. AtlAc, catalytic domain of AtlA.

The structure of the catalytic domain of AtlA (AtlAc) (161 residues) was solved at a resolution of 1.45 Å. It adopts a lysozyme α/β fold with an α-lobe containing five α-helices (α1: 180–197, α2: 202–213; α3: 265–278, α4: 299–306, and α5: 315–327), five helices 3_10_, a small β-hairpin (280–286), and a major β-lobe covering the binding site, made of two antiparallel β-strands (β1: 239–248, β2: 251–260) connecting α2 and α3 (Fig. 1*A*).

This fold superimposes with Auto from *Listeria monocytogenes* with an rmsd of 1.02 Å for 107 Cα atoms and with FlgJ from *Salmonella* Typhimurium (FlgJ_St_) with an rmsd of 2.2 Å for 119Cα, which account for the larger opening of the β-hairpin (Fig. 1, *B* and *D*) (9). The structure also contains five glycerol molecules coming from the cryogenic buffer. Only one polypeptide of AtlAc is found in the asymmetric unit, suggesting that the protein is monomeric in solution.

**Figure 2:**
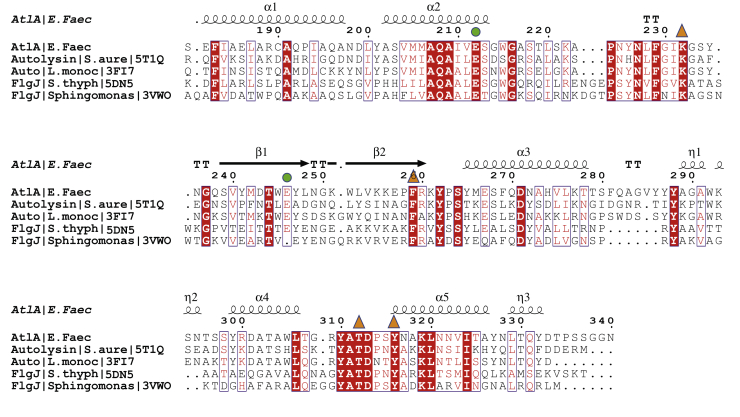
**Structure-based sequence alignment of several GH73 enzymes.** This alignment has been done with the program Expresso (30). AtlAc secondary structures are drawn at the *top* of the alignment. The conserved putative catalytic glutamate residues are shown with *green dots*. The mutated residues used in this study are shown with *orange triangles*. AtlAc, catalytic domain of AtlA; GH73, glycoside hydrolase family 73.

The AtlAc active site shows two highly conserved glutamate residues: E212 is equivalent to *L. monocytogenes* Auto E122 and FlgJ_St_ E184 catalytic residues and is located at a similar position, at the end of helix α1 (Fig. 1*B*). The second predicted catalytic glutamate, E246, stands on β2 and is equivalent to E156 in *L. monocytogenes* Auto and E223 in FlgJ_St_. Unlike Auto and FlgJ_St_, which show an open groove for the active site, the AtlAc active site shapes into a tunnel (Fig. 1*C*). The β-hairpin closing on the α-lobe by forming several hydrogen bonds: E246 OE2 on strand β1 with K232 NZ on a loop between α2 and β1, E246 with T312 OG1 and the water molecule 12 (Wat12), and T312 OG1 with Y247 main chain N (Fig. 1*C*). K232 makes also a bond with OE1 E257 on β2, but this Glu residue is not conserved in the GH73 family (Figs. 1*C* and 2).

Several aromatic residues that have been proposed to be essential for substrate binding or catalysis in GH73 members are also conserved in AtlAc, namely Y310, which belongs to the YATD motif, a signature motif of the GH73 family, Y316, F259, and F229 (Figs. 1*C* and 2) (10).

### PG hydrolysis by AtlA

**Figure 3:**
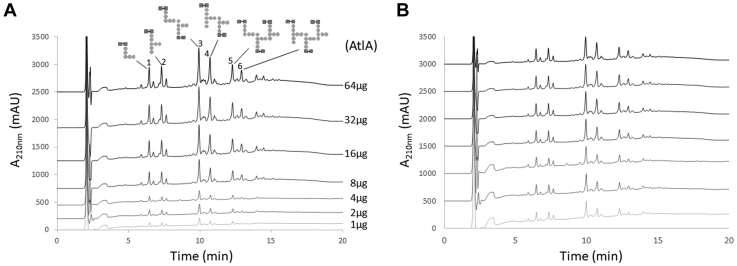
**Reversed-phase HPLC analysis of muropeptides solubilized by AtlA.***A*, *Enterococcus faecalis* peptidoglycan was digested by increasing amounts of recombinant AtlA (from 1 to 64 μg). Soluble fragments released were reduced and separated on a Hypersil C18 column, showing a mixture of monomers and multimers. The schematic structure of major monomers (peaks 1 and 2), dimers (peaks 3 and 4), and trimers (peaks 5 and 6) is shown. GlcNAc and MurNAc are shown as *filled squares*, and peptide stem residues are shown as *filled circles*. *B*, all traces were normalized to display the same amplitude for the major dimer, showing no preferential release of monomeric structures and an overall very similar profile, irrespective of the amount of enzyme used.

The crystal structure of AtlAc revealed a partial occlusion of the catalytic tunnel by the K232 residue (Fig. 1*C*). Based on the analysis of other GH73 catalytic domains, we hypothesized that this residue is flexible to allow the recognition of long glycan chains. An alternative explanation could be that AtlA displays exoglucosaminidase activity, thereby accommodating a smaller substrate in the catalytic tunnel. To test our hypothesis, we investigated whether AtlA displays exoglucosaminidase or endoglucosaminidase activity. Purified PG sacculi were incubated in the presence of increasing amounts of recombinant AtlA, and solubilized muropeptides were analyzed by reversed-phase HPLC. We anticipated that an exoglucosaminidase would release monomeric PG fragments, whilst an endoglucosaminidase activity would release monomeric and multimeric structures simultaneously. The comparison of muropeptide profiles suggested that AltlA displays endoglucosaminidase activity as it generated the same proportion of muropeptides with different oligomerization states, irrespective of the amount of enzyme added (Fig. 3).

### Docking of a PG substrate to the active site of AtlA

**Figure 4:**
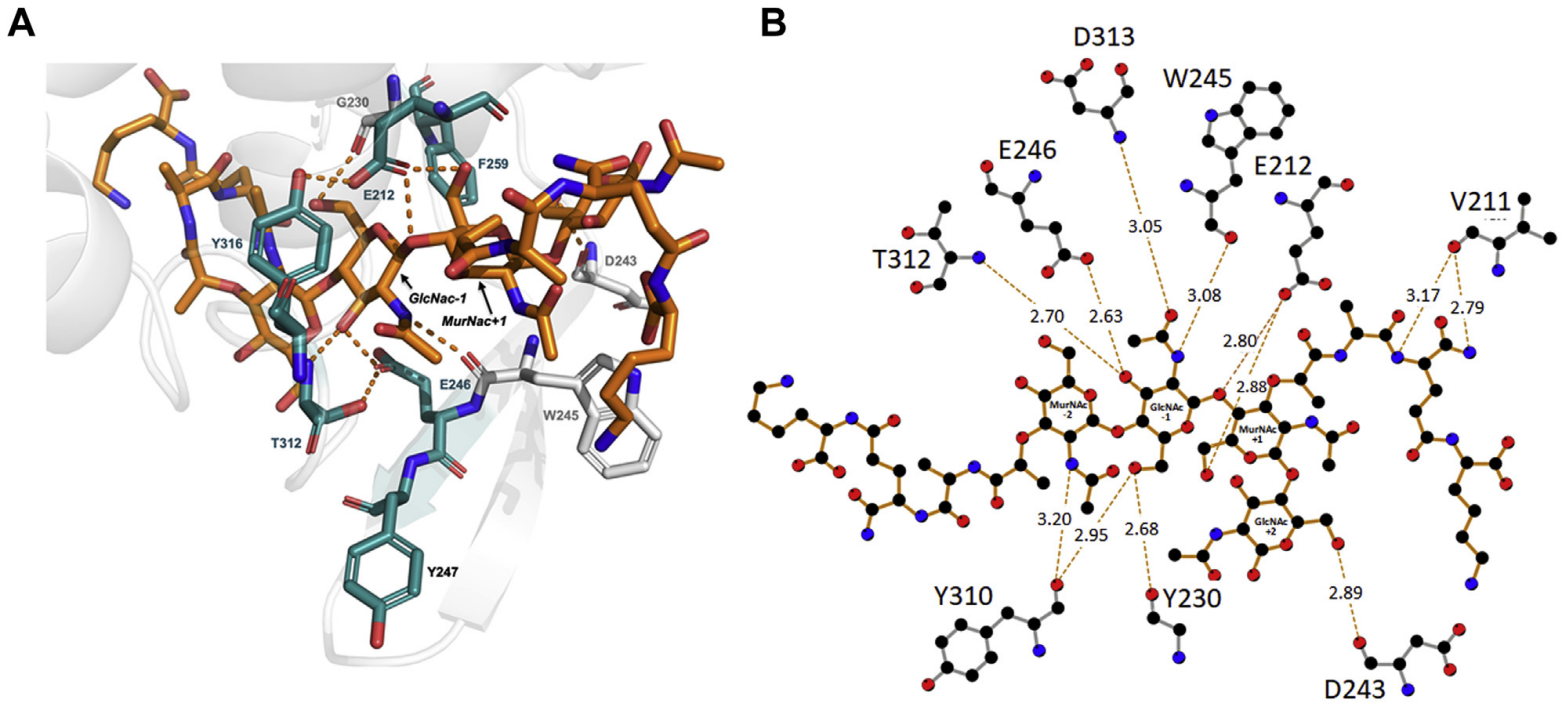
**Model of the interaction between a peptidoglycan (PG) substrate and Atla.***A*, top scoring pose of a PG substrate docked to the active site of Atla. The ligand substrate is represented as *orange sticks*, the receptor is represented as *light gray cartoon*, the main residues of the active site are shown in *sticks*, and the residues selected for mutational studies are highlighted in *blue*. *B*, LigPlot+ representation of the interactions of a PG substrate (ligand) with the active-site residues of Atla (receptor).

Despite several attempts to cocrystallize AtlA catalytic domain with chitooligosaccharides and to soak crystals in ligand solutions, we have not been able to obtain AtlA-GlcNAc cocrystals. To gain insight into AtlA catalytic mechanism and PG binding mode, we therefore docked a PG fragment to the crystal structure of the enzyme. We used a tetrasaccharide chain (MurNAc^−2^–GlcNAc^−1^–MurNAc^+1^–GlcNAc^+2^) with MurNAc residues substituted by short tripeptide stems found in *E. faecalis* PG (l-Ala-γD-Gln-l-Lys) and amenable to docking experiments (Fig. S1). The ligand was docked to a grid that encompasses the putative active site of AtlA and includes the two predicted catalytic glutamates, E212 and E246. The protein was kept rigid except for K232, which blocks part of the active-site tunnel and has to be flexible to accommodate the substrate and allow AtlA endoglucosaminidase activity. The docking produced eight poses for the ligand with binding energies ranging from −6.5 to −5.4 kcal/mol. Figure 4*A* shows the docked complex with the ligand in its best (lowest energy) binding pose. Interestingly, docking experiments using PG ligands with shorter side chains (l-Ala–d-Gln or l-Ala) produced the same pose as for the longer side chain (Fig. S2).

The ligand adopts an extended conformation that spans the active site of AtlA, going through the tunnel that contains the two putative catalytic glutamates. The oxygen of E212 is located 2.8 Å away from the glycosidic bond, in agreement with the cleavage of the glycosidic bond between GlcNAc^−1^ and MurNAc^+1^. The ligand mainly interacts *via* the GlcNAc^−1^ moiety, making several hydrogen bonds with the backbones of G230, W245, Y310, T312, D313, and with the side chain of E246 (Fig. 4*B*), suggesting that the MurNAc^+1^–GlcNAc^+2^ part of the substrate is the first to leave the active site after cleavage. The other sugar moieties of the ligand also establish hydrogen bonds with the protein *via* MurNAc^−2^ (with Y310), MurNAc^+1^ (with E212), and GlcNAc^+2^ (with D243). Although potential interactions may involve the amino acid side chains of the ligands (*e.g.*, with residue V211), the flexibility of the peptide stems renders such predictions unreliable.

### Identification of AtlA residues important for activity *in vitro*

Based on the structural analysis of the AtlA catalytic domain and the docking of a PG fragment, we investigated the role of several residues potentially involved in substrate recognition and catalysis. These included the catalytic glutamate residue E246 that had not been previously studied, K232, F259, T312, and Y316. Y247 was added as a control, as this residue is not expected to contribute to the catalytic activity of AtlA. To conserve the hydrophobic nature, the steric hindrance, or the polar nature of the residues, we mutated F259 into V, T312 into V, Y247 and 316 into S, and E246 into Q. K232 was mutated into A to remove its charge.

**Figure 5:**
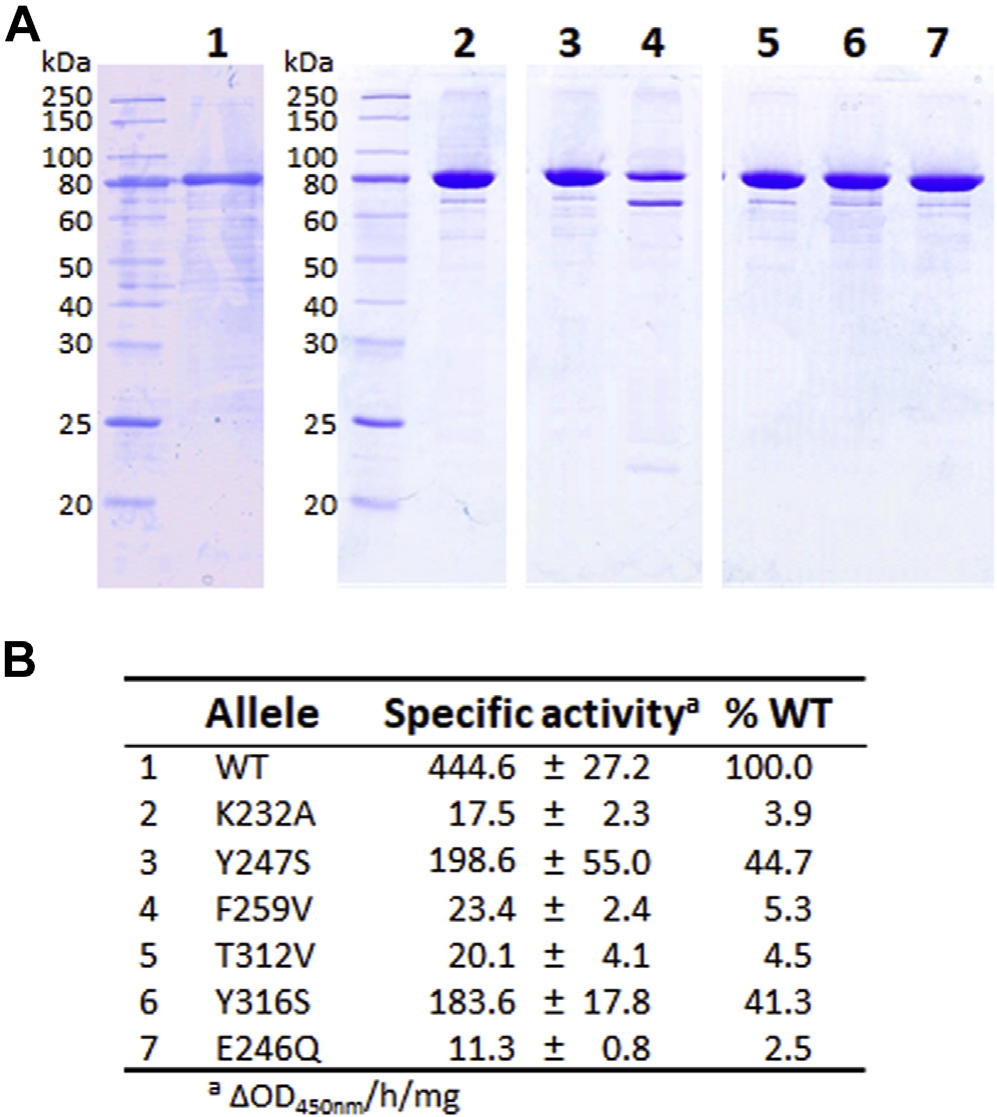
**Purification and characterization of recombinant AtlA enzymatic activity.***A*, SDS-PAGE analysis of full-length recombinant AtlA proteins. AtlA variants were produced in *Escherichia coli* and purified using a one-step immobilized metal affinity chromatography; lane 1, WT; lane 2, K232A; lane 3, Y247S; lane 4, F259V; lane 5, T312V; lane 6, Y316S; and lane 7, E246Q. *B*, specific activity (expressed as Δabsorbance at 450 nm/h/mg) was measured using *Micrococcus luteus* as a substrate.

The expression plasmid encoding full-length recombinant AtlA was mutagenized to introduce these six mutations in the catalytic domain. All recombinant proteins were expressed as soluble proteins and were purified by affinity chromatography (Fig. 5*A*). CD confirmed that the mutations had no major impact on the overall fold of the recombinant proteins (Fig. S3). Using *Micrococcus lysodeikticus* autoclaved cells as a substrate, we determined the specific activity of individual mutants using a spectrophotometric assay (Fig. S4). The Y247S and Y316S mutations had a limited impact on *in vitro*–specific activity (2.2-fold and 2.4-fold decrease, respectively) whilst the four other mutations (K232A, F259V, T312V, and E246Q) had a pronounced impact, leading to residual activities between 2.5% and 5.3% when compared with the WT enzyme (Fig. 5*B*).

### Identification of AtlA residues important for septum cleavage

**Figure 6:**
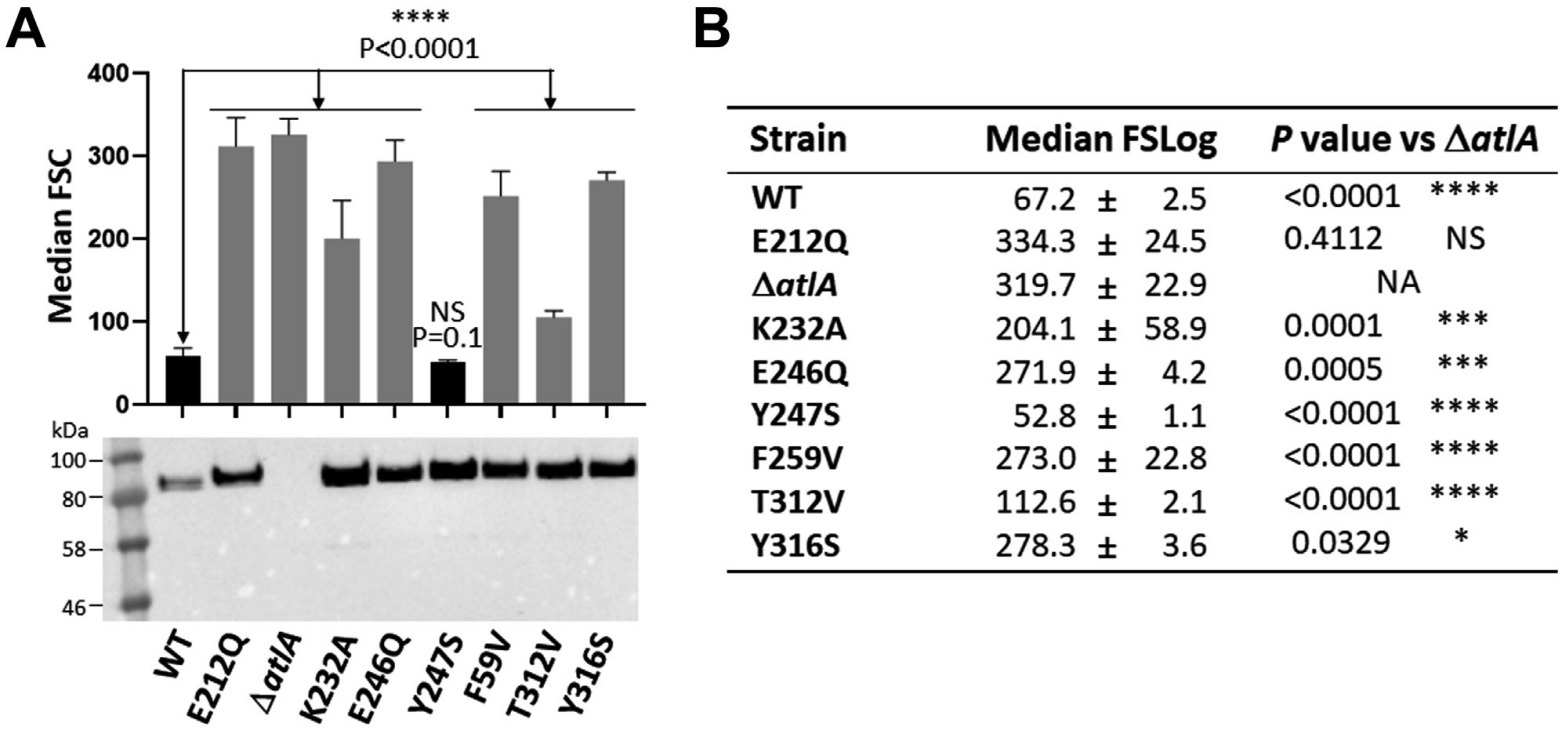
**Flow cytometry analysis of bacterial cell chain length in *Enterococcus faecalis* strains expressing AtlA mutant alleles.***A*, median forward scattered (FSC) light of exponentially growing cells was measured by flow cytometry. The results shown correspond to the average value of six biological replicates for the parental strain (WT), the *atlA* in-frame deletion mutant (Δ*atlA*), and the six strains producing AtlA mutant alleles. All cell chain lengths were significantly different from the WT strain (∗∗∗∗*p* < 0.0001). The amount of protein produced by each strain was checked by Western blot using an anti-AtlA polyclonal serum. The lower intensity in the WT strain is due to a lower amount of material loaded on the gel, as suggested by the absence of signal corresponding to the N-terminal truncation of AtlA (*ca.* 60 kDa). *B*, median FSC values were compared with that from the Δ*atlA* mutant. N/A, not applicable; NS, not significant.

AtlA is a secreted autolysin that undergoes post-translational modifications including proteolytic cleavage and glycosylation (4). To confirm the results obtained with recombinant proteins *in vitro*, we sought to measure the impact of mutations using cell chain length as a readout of enzymatic activity in live bacteria (Fig. 6). Each mutation was introduced on the chromosome by allelic exchange, and the forward scattered light associated with mutant cells was measured by flow cytometry to investigate the capacity of individual AtlA mutant proteins to cleave the septum. All mutant proteins were produced at a similar level, indicating that mutations had very little (if any) impact on stability (Fig. 6*A*). The results were consistent with previous enzymatic assays and the docking model. Y247S has no impact on septum cleavage, confirming that the mutation does not affect the structure of the enzyme. The other five mutations had a clear impact on septum cleavage, forming cell chains significantly longer than the WT (Fig. 6*B*). As expected, E212Q and E246Q mutations had the most pronounced impact on AtlA activity, leading to the formation of extremely long cell chains. Both F259 and K232 were shown to be critical for AtlA activity, whilst T312 had a relatively limited role.

## Discussion

The enzymatic assays using recombinant AtlA and measurement of septum cleavage in strains expressing AtlA variant provided insightful information to propose a catalytic mechanism. E212 is strictly conserved in the GH73 enzyme family and was previously identified as an essential catalytic residue (4). It is positioned at the end of helix α2, on a highly conserved α-lobe and superimposes with E122 from *L. monocytogenes* Auto (9) and E184 from FlgJ_St_ (11) (Fig. 1*B*). AtlA E212 can therefore be confidently identified as the catalytic proton donor. A second glutamate, E246, is positioned on strand β1 like E156 in Auto and E223 in FlgJ_St_ (Fig. 1*B* et D) and has been proposed to be a nucleophile/base catalyst. This second glutamate residue is conserved only in a subgroup of proteobacteria and the bacteroidetes as mentioned in the phylogenetic study that classified the GH73 sequences into five different clusters (10). In clusters 1 and 2, GH73 enzymes possess a β-lobe that folds into an extended β-hairpin that contributes to shaping the active site and harbors a conserved glutamate (10). This β-hairpin has been modeled in Auto, FlgJ_St_, and more recently in *Sphingomonas* sp. FlgJ but is often incomplete in other structural homologs like TM0633 because of its flexibility (9, 10, 11, 12). In Auto and FlgJ_St_ structures, the position of the β-hairpin places the two catalytic glutamates at 13 and 20 Å apart, respectively, and this is due to the presence of an α-helix or a β-strand of a neighboring symmetry monomer that occludes their active sites (9, 11, 12) (Fig. 1*B*).

**Figure 7:**
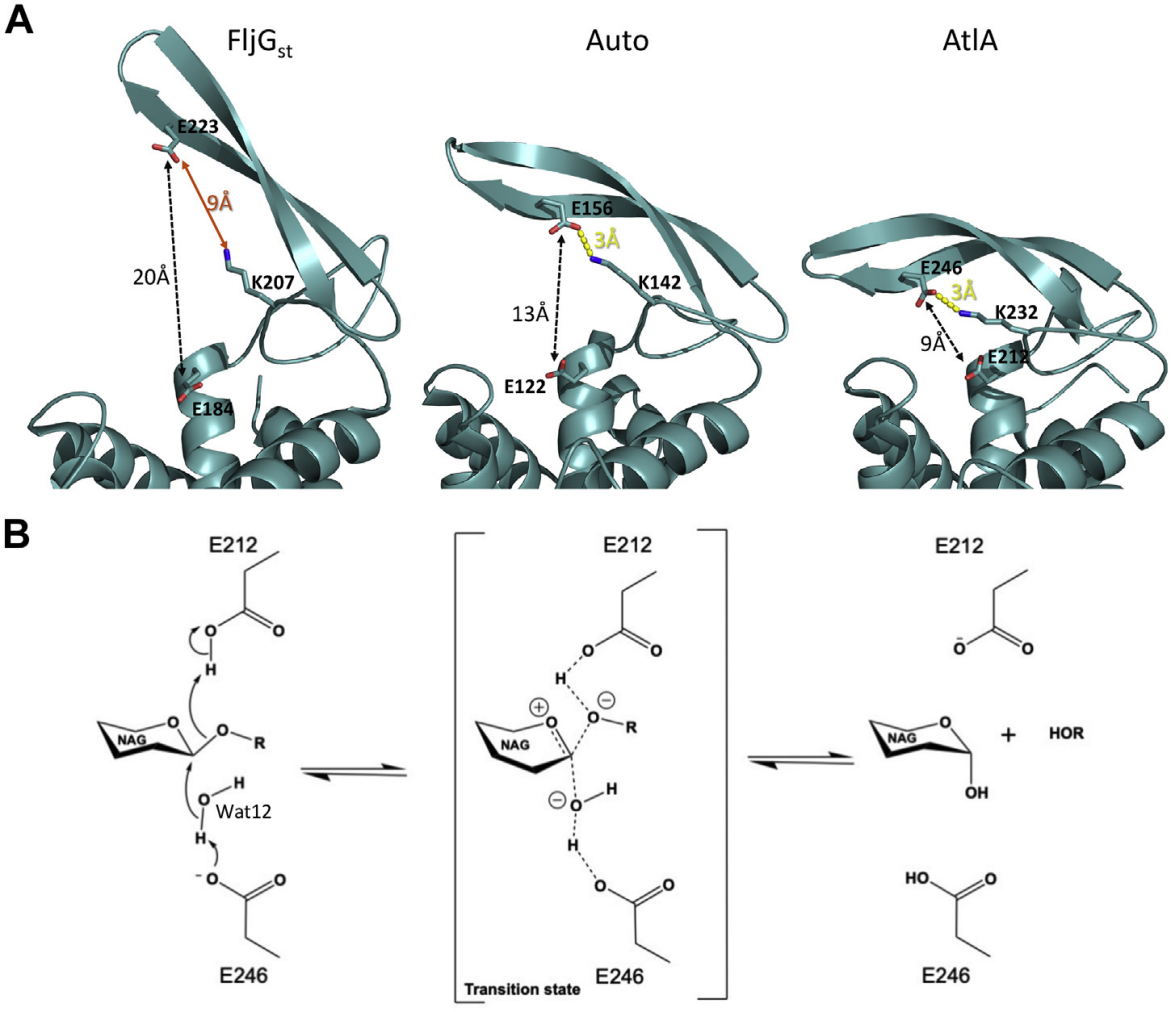
**AtlA catalytic mechanism.***A*, the motion performed by the β-hairpin to close on the active site is shown by comparing FlgJ_St_, Auto, and AtlA β-hairpin's positions. In AtlA and Auto, K232 and K142 make electrostatic bonds with the nucleophiles E246 and E156, respectively (*yellow dashed lines*). In FlgJ_St_, K207 is too far to bind to E223; the distance is shown with an *orange arrow*. *B*, AtlA is an *N*-acetylglucosaminidase hydrolyzing the β-1,4 linkage between NAG and MurNAc using an inverting mechanism. E246 (catalytic base) activates Wat12 by abstracting a proton, which enables the water molecule to make a nucleophilic attack on the anomeric carbon and leads to the formation of an oxocarbenium ion–like transition state. E212 plays the role of an acid that will donate a hydrogen to the scissile glycosidic bond. This glycolysis produces an α-anomeric configuration of the NAG at the reducing end. NAG, *N*-acetylglucosamine; Wat12, water molecule 12.

In these structures, the β-hairpin is widely open, and the authors suggest that the β-hairpin takes this position to accommodate the substrate in the active site. In AtlAc, the β-hairpin shows a “closed” conformation and the distance between E212 and E246 is 9 Å, which is the distance required for a classic type 2 nucleophile substitution with an inverting mechanism (13) (Fig. 7*A*). We, therefore, propose that E212 acts as a general acid to protonate the glycosidic oxygen, favoring the aglycon departure, while the general base E246 activates a water molecule to make a nucleophile attack on the sugar anomeric carbon. E246 OE1 is making a hydrogen bond with a Wat12 and K232 (Fig. 7*B*). This water molecule could therefore be activated by E246 if the latter encounters a displacement of a negative charge and takes a proton from Wat12, which will, in turn, be able to perform the nucleophilic attack on the anomeric carbon of the substrate. In previous studies where structural homologs show a β-hairpin widely open, the authors proposed that the β-hairpin will undergo several conformational changes to accommodate the substrate in the active site and subsequently place the two catalytic glutamate residues at the proper distance for hydrolysis of the glycosidic bond (13). Our crystallographic data strongly support this model and suggest a contribution of a conserved lysine residue (K232) in this process, as demonstrated by the loss of AtlA activity associated with the K232 mutation both *in vitro* and *in vivo*. The structures of Auto and FlgJ_St_ illustrate intermediary positions of the β-hairpin. In the widely open active site of FlgJ_St_, K207 is too far to contact E223, whereas in Auto, K142 can bind to the catalytic E156 and make electrostatic contact to initiate the closing of the β-hairpin. Our AtlAc crystal structure shows the electrostatic bond between K232 and E246 maintaining the position of the β-hairpin resulting in a closed active site (Fig. 7*A*). We propose that this electrostatic contact between the K232 and the E246 stabilizes the charges to help its attack on the water molecule. The identification of the endoglucosaminidase activity together with modeling of the tetrasaccharide peptide bound in the active site indicates that after the movement of the β-hairpin, K232 adopts another conformation to allow substrate binding and catalysis. Collectively, our results indicate that AtlA uses an inverting mechanism to hydrolyze the β-1,4 glycosidic bond.

## Experimental procedures

### Bacterial strains, plasmids, and growth conditions

All strains and plasmids used in this study are described in Table S1. The bacteria were grown at 37 °C in brain heart infusion (BHI) broth or agar (15 g/l) (Difco Laboratories). When required, *Enterococcus coli* was grown in the presence of 100 μg/ml ampicillin (for protein expression) or 200 μg/ml erythromycin (for pGhost selection). *E. faecalis* transformants were selected with 30 μg/ml erythromycin.

### Plasmid construction

pML420 was built to express AtlA catalytic domain. The DNA fragment encoding amino acids 172 to 337 was PCR amplified from V583 genomic DNA (14) with oligonucleotides EF Atla_cat_F and Atla_cat_R (Table S1) using Vent DNA polymerase (NEB). The resulting fragment was cloned in frame with the hexahistidine sequence of pET2818, a pET2816b derivative (8), using NcoI and BamHI. Plasmids expressing full-length AtlA derivatives (with single amino-acid substitutions) were constructed by site-directed mutagenesis using plasmid pML118 as a template (8) following the QuikChange mutagenesis protocol (15) and oligonucleotides described in Table S1. pML118 derivatives were used as a template to amplify the DNA fragments encoding atlA alleles. The corresponding PCR products were cloned in pGhost9 using XhoI and EcoRI.

### Construction of *E. faecalis* mutants by allele exchange

The protocol described previously was followed (16). *E. faecalis* was electroporated with pGhost9 derivatives, and transformants were selected at 30 °C in the presence of erythromycin. Single crossingovers were induced at nonpermissive temperature (42 °C) and screened by PCR. The second recombination event was triggered by subculturing recombinant clones in BHI at 42 °C. Erythromycin-sensitive colonies were screened by PCR to identify mutants.

### Production and purification of recombinant proteins

*E. coli* Rosetta(DE3) pLysS cells (Novagen) containing recombinant pET vectors were cultured in ZY autoinduction medium (17) containing 100 mg/ml of ampicillin and 34 mg/ml of chloramphenicol at 37 °C until the absorbance reaches 0.6 at 600 nm. The cultures were then incubated overnight at 17 °C. Cells were harvested by centrifugation, and the frozen pellets were resuspended in lysis buffer (50 mM Tris–HCl [pH 8], 300 mM NaCl, 10 mM imidazole, 1 mM EDTA, 0.25 mg/ml lysozyme, 5% glycerol, and 0.1% Triton). After a 30 min of incubation with 0.1 mg/ml deoxyribonuclease and 0.02 M magnesium sulphate at 4 °C, the cells were disrupted by sonication on ice. The lysate was clarified by centrifugation, and the supernatant was applied onto a nickel-chelate affinity resin using an ÄktaXpress (Cytiva). The resin was washed with 50 mM Tris–HCl (pH 8), 300 mM NaCl, and 50 mM imidazole. The protein was eluted with 50 mM Tris–HCl (pH 8), 300 mM NaCl, and 250 mM imidazole and dialyzed against the gel filtration buffer (20 mM Mes [pH 6], 100 mM NaCl, and 1 mM DTT). The protein was further purified on a Superdex 75 16/60 column (Cytiva). Pure fractions, as analyzed by SDS-PAGE were pooled.

### CD spectroscopy

CD samples were diluted to a concentration of 0.25 mg/ml in 25 mM Tris–HCl + 150 mM NaCl buffer, pH 8.0. Far-UV CD spectra were acquired with a Jasco J-810 spectropolarimeter using a 0.1 cm pathlength cell. Spectra were acquired with a Peltier thermally controlled cuvette holder at 25 °C. Spectra were recorded from 190 to 350 nm, with a data pitch of 1 nm at a rate of 50 nm/min, a response time of 8 s, and averaged over five accumulations (18).

### Crystallization, data collection, and processing

AtlAc was concentrated to 14 mg/ml using a 10 kDa cutoff ultracentrifugation membrane (Thermo Fisher Scientific). Crystals were obtained at 20 °C by screening The Wizard Classic crystallization kit (Molecular Dimensions) using a nanoliter sitting drops setup with automated crystallization TECAN Genesis and ttplabtech Mosquito robots. A total of 300 μl of AtlAc (14 mg ml/1) was mixed to 100 μl of reservoir solution composed of 0.2 M magnesium chloride, 10% PEG 3000 and 0.1 M sodium cacodylate (pH 6.5). A single crystal of AtlAc was mounted on a rayon loop, soaked in a cryoprotectant solution composed of the crystallization conditions with 23% glycerol, and subsequently flash-cooled to 120 K. A dataset was collected at 1.4 Å at the ID30B beamline (ESRF). Data were integrated, scaled, and reduced with XDS (19) and AIMLESS (20). Unless otherwise cited, all further crystallographic computations were carried out using the CCP4 suite of programs (20).

### Phasing, model building, and refinement

The structure was solved by molecular replacement, using MolRep (21), with a search model of AtlAc built by Modeller using Auto structure from *L. monocytogenes* (54% sequence identity with AtlAc) as a template (Protein Data Bank code: 3FI7) (9, 22). The solution of MolRep gave a correlation coefficient of 0.47 for one monomer in the asymmetric unit. The electron density map calculated from the model was of sufficient quality to allow tracing of 99% of the molecule (156 residues) using the REFMAC/ARP-wARP programs (23, 24). Unless otherwise cited, all further crystallographic computations were carried out using the CCP4 suite of programs (20). The model was refined using REFMAC (23) with manual correction using Coot (25). The final model contains 1281 nonhydrogen protein atoms with 182 water molecules and five glycerol molecules. The crystallographic *R*_cryst_ and *R*_free_ values are 14 and 17%, respectively (Table 1).

**Table 1:** Structural data collection and refinement statistics

Data	AtlAc
Crystal parameters	
Space group	P3_1_21
Cell parameters (Å)	*a* = *b* = 62.55, *c* = 93.22
Data quality	
Wavelength (Å)	0.9762 (PX1)
Resolution of data (Å)	46.8–1.45
Unique reflections	38,042
*R*_merge_ (outer shell)^a^	0.06 (0.93)
Mean *I*/sigma (*I*) (outer shell)	9.8 (1.1)
Completeness (outer shell) %	99.9 (99.9)
CC1/2 (outer shell)	0.99 (0.76)
Multiplicity (outer shell)	4.7 (4.2)
Mean *B*-factor (Å^2^)	20.3
Refinement	
*R*_cryst_^b^	0.14
*R*_free_^c^	0.17
rmsd 1–2 bonds (Å)	0.012
rmsd 1–3 angles (°)	1.539
Ramachandran plot	
Favored	98%
Outliers	0%
Protein Data Bank code	7QFU

a *R*_merge_ = (S_*hkl*_ S_*i*_½*I*_*hkl*_ − [*I*_*hkl*_]½/S_*hkl*_ S_*i*_ [*I*_*hkl*_]).

b *R*_cryst_ = S_*hkl*_| |*F*_o_| − |*F*_c_| |/S_*hkl*_*|F*_o_|.

c *R*_free_ is calculated for randomly selected reflections excluded from refinement.

### Docking experiments

Molecular docking was performed in Autodock Vina (26). The structure of Atla was used as a docking model. Ligand and receptor structures were prepared in JLigand (27) and AutoDockTools (28). Partial charges were calculated using Gasteiger–Hückel method. Exhaustiveness was set to eight, and default parameters were used unless otherwise stated. The ligand was treated as flexible, and the protein was kept rigid during the docking runs, except for the side chain of K232, which was treated as flexible. The grid size was set to 22 × 30 × 24 (Å), and the grid box's center points were set to target the active site of the protein, with the center at X = 32, Y = 20, and Z = −11.5. All 3D and 2D representations of protein–ligand complexes were visualized using PyMOL (The PyMOL Molecular Graphics System) and LigPlot+ (29), respectively.

### Determination of PG hydrolase activity

Hydrolysis of purified cell walls was measured using an Ultrospec 2000 spectrophotometer (Amersham Biosciences) and following the decrease in turbidity at 450 nm for 15 min at 37 °C in 25 mM Tris–HCl, pH 7.5, and 100 mM NaCl buffer. PG was resuspended at a concentration giving an absorbance at 450 nm of 0.6. Various dilutions of AtlA and its derivatives were tested to identify conditions in which the velocity of hydrolysis was proportional to enzyme concentration. Enzymatic activity was expressed as absorbance at 450 nm units per min per mmol of protein; the residual activity of individual mutants was expressed as the percentage of WT activity.

For zymogram analysis, crude extracts were separated by SDS-PAGE using gels containing 0.2% autoclaved *M. lysodeikticus* cells. After electrophoresis, the proteins were renatured by incubating the gel for 24 h in 25 mM Tris (pH 8.0) buffer containing 0.1% Triton at 37 °C. Lytic activities could be visualized as clear bands on the opaque SDS-PAGE.

### Western blot analyses

Cells were grown in BHI to exponential phase. The equivalent of 250 μl of acid-washed <106 μm glass beads (SIGMA) was added to a 1.5 ml aliquot of the culture, and cells were broken using a Fastprep homogenizer (MP Biomedicals) with six cycles at maximum speed with 1 min pause between cycles. The equivalent of 25 μl of culture was loaded on a 12% SDS-PAGE and transferred to nitrocellulose. AtlA was detected using a rabbit polyclonal antibody against the full-length protein as previously described (4).

### Flow cytometry and statistical analyses

Overnight static cultures at 37 °C were diluted 1:100 into fresh broth (absorbance of ∼0.02 at 600 nm) and grown to midexponential phase (absorbance of ∼0.2–0.4 at 600 nm). Bacteria were diluted 1:100 in filtered phosphate-buffered saline and analyzed by flow cytometry using a Millipore Guava easyCyte H2L system. Light scatter data were obtained with logarithmic amplifiers for 20,000 events. Forward scattered light values were compared using GraphPad (GraphPad Software, Inc) and an unpaired *t* test with Welch's correction.

## Data availability

All data presented in this article are to be shared upon request to Dr Florence Vincent (fvincent.cnrs@univ-amu.fr) or Dr Stéphane Mesnage (s.mesnage@sheffield.ac.uk).

The atomic coordinates of AtlAc were deposited in the Protein Data Bank (code: 7QFU).

## Supporting information

This article contains supporting information (Figs. S1–S4 and Table S1).

## Conflict of interest

The authors declare that they have no conflicts of interest with the contents of this article.
