# Supplementary material for: Molecular basis for substrate recognition and septum cleavage by AtlA, the major N-acetylglucosaminidase of Enterococcus faecalis
Source: J Biol Chem. 2022 Apr 7;298(5):101915. doi: 10.1016/j.jbc.2022.101915 (PMC9108991; doi:10.1016/j.jbc.2022.101915)
Supplement: Supplemental Figures S1–S4 and Table S1 [file mmc1.pdf]

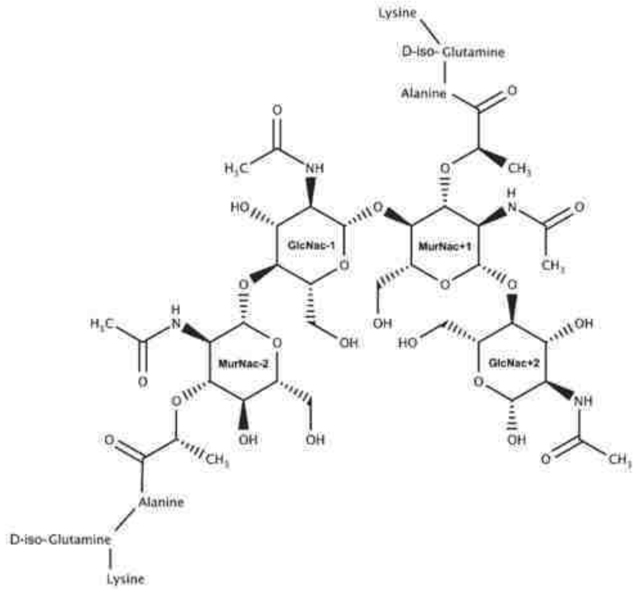

Figure S1. 2D representation of the peptidoglycan substrate used in the docking experiment. The molecule is a repetition of beta-1,4-linked *N*-acetylmuramic acid (MurNAc) and *N*-acetylglucosamine (GlcNAc) residues, with MurNAc residues substituted by tripeptide stems made of L-Alanine, D-isoglutamine and L-Lysine.

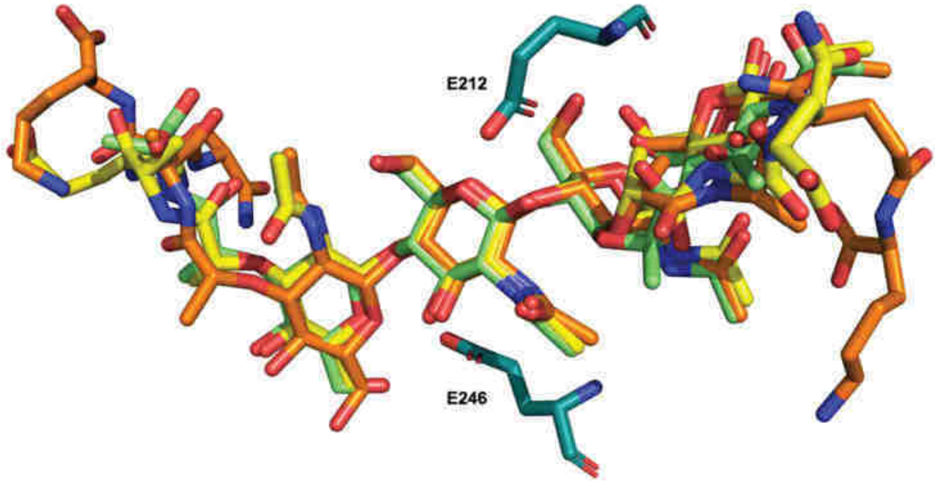

Figure S2. Superposition of the docking poses for 3 peptidoglycan substrates with various peptide chain lengths. A tripeptide (L-alanine, D-isoglutamine, L-lysine) is shown in orange sticks, a dipeptide (L-alanine, D-isoglutamine) in yellow sticks and a monopeptide (L-alanine) in green sticks. The two putative catalytic glutamates are shown as blue sticks.

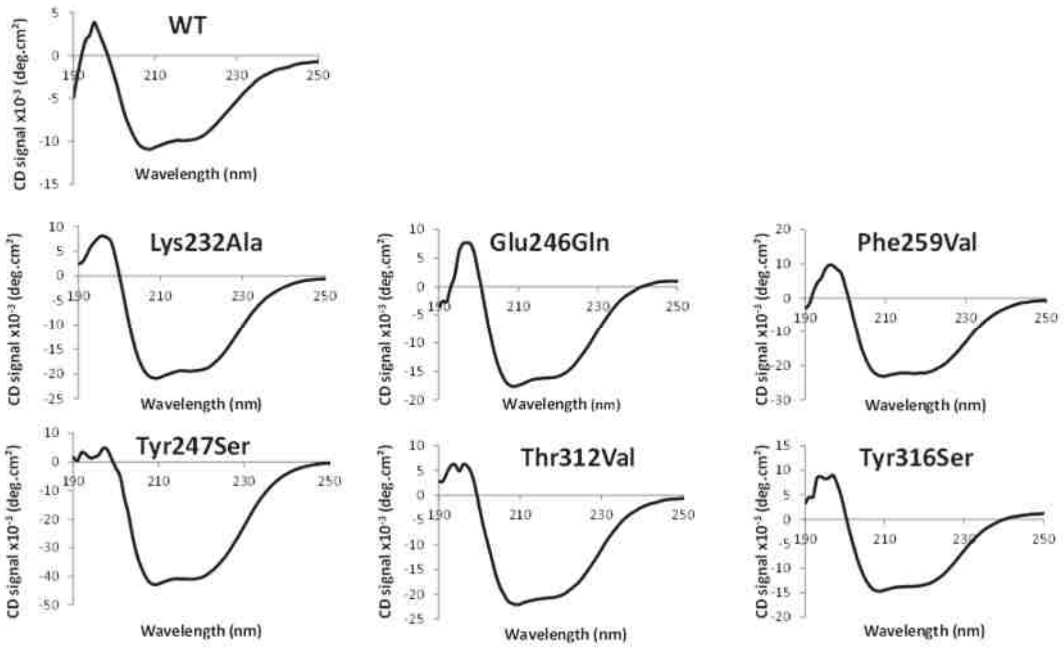

Figure S3. Circular dichroism analysis of recombinant AtlA variants. Each protein was resuspended at a concentration of 0.25 mg/ml and far-UV CD spectra were acquired with a Peltier thermally controlled cuvette holder at 25°C, from 190 to 350 nm.

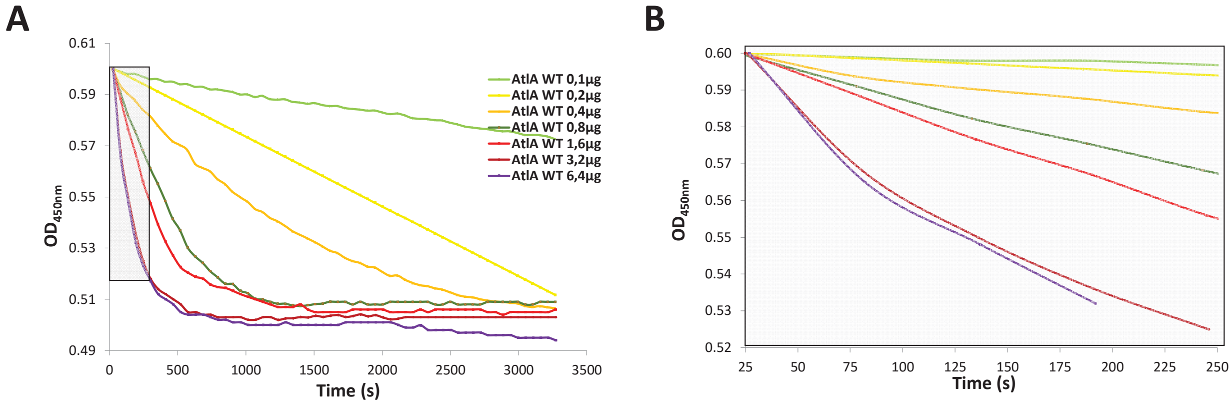

Figure S4. Determination of AtlA specific activity. A, *M. luteus* autoclaved cells were incubated at 37°C in the presence of various amounts of recombinant AtlA and the optical density at 450nm (OD_{450nm}) was recorded over time. **B**, The specific activity (expressed in ΔOD_{450nm}/h/mg of protein was calculated from the linear portion of the curve.

Table S1. Bacterial strains, plasmids and oligonucleotides.

Strains/plasmids/ oligonucleotides	Relevant properties	Source
**Strains**		
Enterococcus faecalis		
V583	Sequenced strain (clinical isolate)	(14)
Escherichia coli		
BL21(DE3)	Expression strain	NEB
NEB5alpha	Cloning strain (pET derivatives)	NEB
TG1 (*repA*$^{+}$)	Cloning strain (pGhost derivatives)	Lab stock
Micrococcus lysodeikticus	Substrate for zymograms	ATCC4698
**Plasmids**		
pET2818	pET28/16 variant for C-terminal Histidine-tag fusion	Lab stock
pGhost9	Thermosensitive plasmid (ErmR)[a] for allele replacement	(16)
pML420	pET2818 derivative expressing AtlA catalytic domain (residues 172 to 336)	This work
pML118	pET2818 derivative expressing full length AtlA (residues 54 to 737)	(8)
pMLE212Q	pML118 derivative with the D313N mutation	This work
pMLK232A	pML118 derivative with the K232A mutation	This work
pMLE246Q	pML118 derivative with the D313N mutation	This work
pMLF259V	pML118 derivative with the F259V mutation	This work
pMLT312V	pML118 derivative with the T312V mutation	This work
pMLY316S	pML118 derivative with the D313N mutation	This work
pGE212Q	pGhost9 derivative to build mutant E212Q	(4)
pGK232A	pGhost9 derivative to build mutant K232A	This work
pGE246Q	pGhost9 derivative to build mutant E246Q	This work
pGF259V	pGhost9 derivative to build mutant F259V	This work
pGT312V	pGhost9 derivative to build mutant T312V	This work
pGY316S	pGhost9 derivative to build mutant Y316S	This work
**Oligonucleotides**		
Atla cat F	ATACCATGGGGTCAGCGTTATCACCGACGC	
Atla cat R	ATGGGATCCAGAAGATGGTGTATCATATTGAGTTAAG	
AtlA pG F	AAAGAATTCACAGAAGAGCAGCCAACAAATGC	
AtlA pG R	ATTCTCGAGACCAACTTTTAAAGTTTGACCAATATAAATTG	
E212Q F	GATGATGGCTCAAGCAATCGTTCAAAGTGGTTGGGGAGCAAGTA	
E212Q R	CGTACTTGCTCCCCAACCACTTTGAACGATTGCTTGAGCCATCAT	
K232A F	CTTATTTGGGATTGCAGGCAGCTACAATGGACAATCTGTC	
K232A R	TTGTAGCTGCCTGCAATCCCAAATAAGTTATAGTTTGGTG	
E246Q F	CTATATGGATACATGGCAATATTTAAACGGCAAATGGTTAGTG	
E246Q R	TTGCCGTTTAAATATTGCCATGTATCCATATAGACAGATTGTC	
F259V F	GAAAAAAGAACCTGTCCGTAAATATCCTTCTTACATGGA	
F259V R	GATATTTACGGACAGGTTCTTTTTTCACTAACCATTT	
T312V F	GGTCGTTATGCGGTAGATCCTAGCTACAATGCTAAATTA	
T312V R	GTAGCTAGGATCTACCGCATAACGACCTGTTAACCAAGCA	
Y316S F	CAGATCCTAGCTCCAATGCTAAATTAAATAATGTCATT	
Y316S R	AATTTAGCATTGGAGCTAGGATCTGTCGCATAACGACC	

[a] ErmR, resistance to erythromycin
